# Supplementary material for: Neutrophil-mediated oxidative stress and albumin structural damage predict COVID-19-associated mortality
Source: eLife. 2021 Nov 25;10:e69417. doi: 10.7554/eLife.69417 (PMC8641949; doi:10.7554/eLife.69417)
Supplement: Supplementary file 1. [file elife-69417-supp1.pptx]

## Slide 1
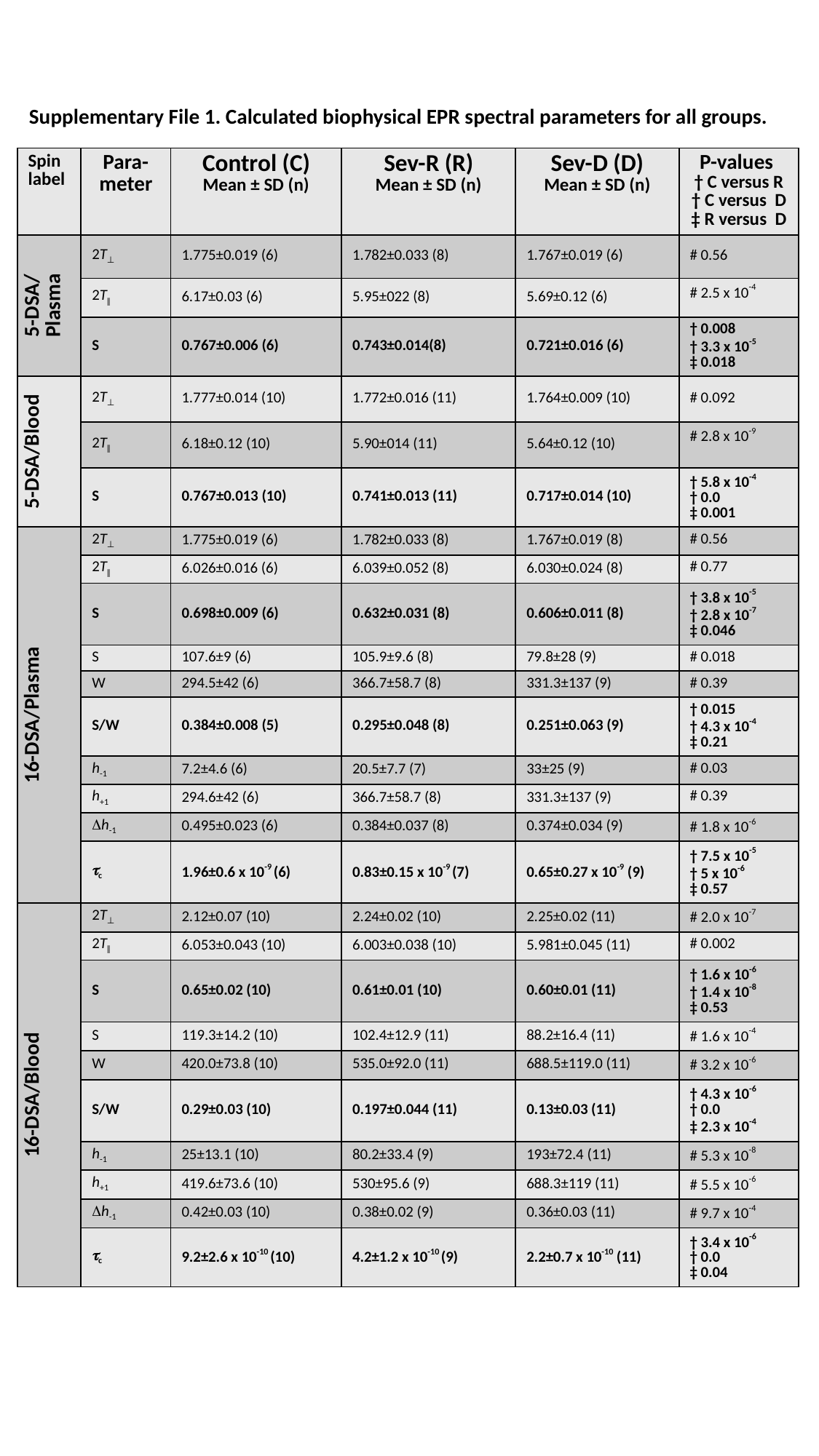

Supplementary File 1. Calculated biophysical EPR spectral parameters for all groups.
| Spin label | Para-meter | Control (C) Mean ± SD (n) | Sev-R (R) Mean ± SD (n) | Sev-D (D) Mean ± SD (n) | P-values † C versus R † C versus D ‡ R versus D |
| --- | --- | --- | --- | --- | --- |
| 5-DSA/Plasma | 2T⊥ | 1.775±0.019 (6) | 1.782±0.033 (8) | 1.767±0.019 (6) | # 0.56 |
| | 2T‖ | 6.17±0.03 (6) | 5.95±022 (8) | 5.69±0.12 (6) | # 2.5 x 10^-4^ |
| | S | 0.767±0.006 (6) | 0.743±0.014(8) | 0.721±0.016 (6) | † 0.008 † 3.3 x 10^-5^ ‡ 0.018 |
| 5-DSA/Blood | 2T⊥ | 1.777±0.014 (10) | 1.772±0.016 (11) | 1.764±0.009 (10) | # 0.092 |
| | 2T‖ | 6.18±0.12 (10) | 5.90±014 (11) | 5.64±0.12 (10) | # 2.8 x 10^-9^ |
| | S | 0.767±0.013 (10) | 0.741±0.013 (11) | 0.717±0.014 (10) | † 5.8 x 10^-4^ † 0.0 ‡ 0.001 |
| 16-DSA/Plasma | 2T⊥ | 1.775±0.019 (6) | 1.782±0.033 (8) | 1.767±0.019 (8) | # 0.56 |
| | 2T‖ | 6.026±0.016 (6) | 6.039±0.052 (8) | 6.030±0.024 (8) | # 0.77 |
| | S | 0.698±0.009 (6) | 0.632±0.031 (8) | 0.606±0.011 (8) | † 3.8 x 10^-5^ † 2.8 x 10^-7^ ‡ 0.046 |
| | S | 107.6±9 (6) | 105.9±9.6 (8) | 79.8±28 (9) | # 0.018 |
| | W | 294.5±42 (6) | 366.7±58.7 (8) | 331.3±137 (9) | # 0.39 |
| | S/W | 0.384±0.008 (5) | 0.295±0.048 (8) | 0.251±0.063 (9) | † 0.015 † 4.3 x 10^-4^ ‡ 0.21 |
| | h-1 | 7.2±4.6 (6) | 20.5±7.7 (7) | 33±25 (9) | # 0.03 |
| | h+1 | 294.6±42 (6) | 366.7±58.7 (8) | 331.3±137 (9) | # 0.39 |
| | Dh-1 | 0.495±0.023 (6) | 0.384±0.037 (8) | 0.374±0.034 (9) | # 1.8 x 10^-6^ |
| | tc | 1.96±0.6 x 10^-9^ (6) | 0.83±0.15 x 10^-9^ (7) | 0.65±0.27 x 10^-9^ (9) | † 7.5 x 10^-5^ † 5 x 10^-6^ ‡ 0.57 |
| 16-DSA/Blood | 2T⊥ | 2.12±0.07 (10) | 2.24±0.02 (10) | 2.25±0.02 (11) | # 2.0 x 10^-7^ |
| | 2T‖ | 6.053±0.043 (10) | 6.003±0.038 (10) | 5.981±0.045 (11) | # 0.002 |
| | S | 0.65±0.02 (10) | 0.61±0.01 (10) | 0.60±0.01 (11) | † 1.6 x 10^-6^ † 1.4 x 10^-8^ ‡ 0.53 |
| | S | 119.3±14.2 (10) | 102.4±12.9 (11) | 88.2±16.4 (11) | # 1.6 x 10^-4^ |
| | W | 420.0±73.8 (10) | 535.0±92.0 (11) | 688.5±119.0 (11) | # 3.2 x 10^-6^ |
| | S/W | 0.29±0.03 (10) | 0.197±0.044 (11) | 0.13±0.03 (11) | † 4.3 x 10^-6^ † 0.0 ‡ 2.3 x 10^-4^ |
| | h-1 | 25±13.1 (10) | 80.2±33.4 (9) | 193±72.4 (11) | # 5.3 x 10^-8^ |
| | h+1 | 419.6±73.6 (10) | 530±95.6 (9) | 688.3±119 (11) | # 5.5 x 10^-6^ |
| | Dh-1 | 0.42±0.03 (10) | 0.38±0.02 (9) | 0.36±0.03 (11) | # 9.7 x 10^-4^ |
| | tc | 9.2±2.6 x 10^-10^ (10) | 4.2±1.2 x 10^-10^ (9) | 2.2±0.7 x 10^-10^ (11) | † 3.4 x 10^-6^ † 0.0 ‡ 0.04 |
